# New Treatment Strategy for a Delayed Skin Necrosis Related to a Calcaneal Fracture

**DOI:** 10.2174/1874325001711011568

**Published:** 2017-12-29

**Authors:** Konstantinos C. Xarchas, George Kyriakopoulos, Maria Vlachou, Katerina Manta

**Affiliations:** Department of Orthopaedics and Trauma, Athens General Hospital G. Gennimatas, Athens, Greece

**Keywords:** Calcaneum, Avulsion fracture, Skin necrosis, Infection, Treatment, PRP, VAC

## Abstract

In calcaneal avulsion fractures, skin necrosis as a result of pressure from the underlying fragment is a fairly common and serious complication. In spite of proper treatment, skin healing complication may occur. We report a calcaneal fracture case complicated by skin necrosis and managed with a novel treatment strategy including application of Platelet Rich Plasma (PRP) and Vacuum Assisted Closure (VAC). This is the first application of combined PRP and VAC treatment in a calcaneal fracture complicated by skin necrosis and was accompanied with very favorable outcomes while avoiding other more complex treatment modalities.

## 1 INTRODUCTION

The management of calcaneus fractures and their associated soft tissue injuries, is a challenging task for the surgeon and the use of non-operative *versus* operative interventions for their treatment remains a controversial topic. Surgery for calcaneus fractures should be ideally delayed for 10-14 days, especially in the presence of significant edema or fracture blister formation. Exceptions to this rule include open fractures, the presence of a compartment syndrome in the foot and calcaneal avulsion fractures, which should prompt immediate surgery for appropriate intervention [1]. Avulsion fractures often occur in elderly women with osteoporosis and are classified into three types: The type I fracture is the most common calcaneal avulsion fracture (40%).A type I fracture is a true avulsion type, called a “sleeve” fracture; a type II fracture is an “open beak” type; and a type III fracture is a type of infrabursal avulsion of the middle third of the tuberosity [2, 3] Fig. (**1**). A review of the relevant literature supports that a delayed approach of these fractures can lead to skin necrosis and severe wound complications. It should be noted that the soft tissue overlying the Achilles tendon and calcaneal tuberosity is thin, with a precarious blood supply and for that reason these fractures should be treated as emergencies with open reduction and internal fixation. Signs of skin at risk at presentation, are blanching and lack of capillary refill [4]. These are followed by overt skin necrosis and bony exposure as in our case. Except skin necrosis, complications after non-operative treatment of calcaneal tuberosity fractures, include Haglund’s deformity and loss of plantar-flexion power [4].

## 2 CASE REPORT

### 2.1 Case Presentation

A 75 year old lady, felt excruciating pain on her right heel cord when she half -stepped on a pavement, over-flexing her right foot in an attempt to avoid a car. She was put in a back slab by her primary care physician and referred to our hospital where she presented 24 hours after the incident. On examination, there was swelling and bruising on the back side of the heel, but no skin blistering. Her right ankle plantar flexion power was markedly diminished. No ankle plantar flexion was demonstrated upon squeeze of her right calf. A bone fragment was palpable just under the skin above the heel bump. X-rays confirmed an avulsion fracture of the heel at the insertion of the Achilles tendon Fig. (**2**). Urgent operative treatment was the only option for this displaced (type 2) fracture of the calcaneal tuberosity, in order to restore continuity of the gastrosoleus complex and avoid skin necrosis due to pressure by the displaced fragment. The patient’s past medical history was significant only for hypertension and hyperlipidemia.

### 2.2 Operative Findings

**Fig. (3):**
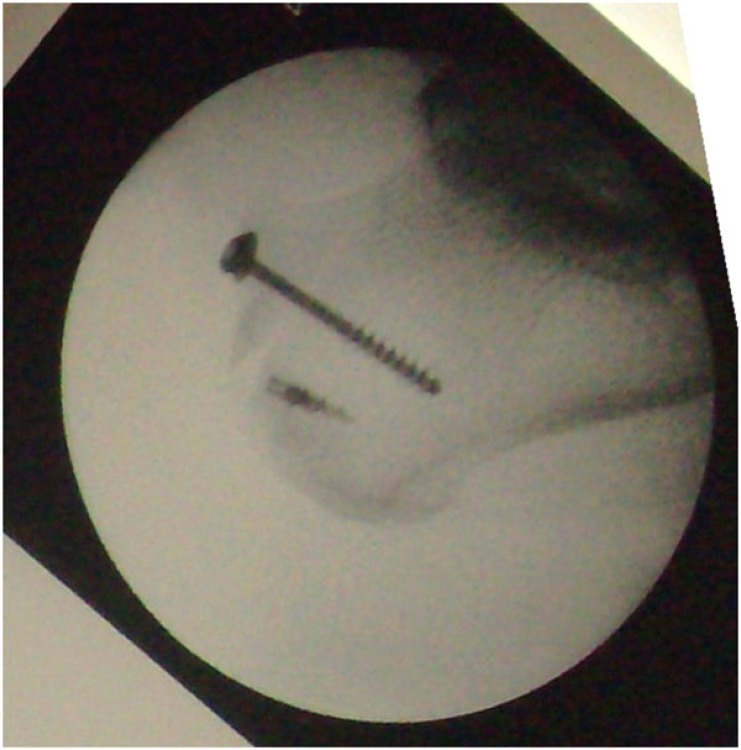
Lateral x-ray after open reduction and internal fixation of avulsed fragment.

Under general anesthesia, with the patient in a prone position and using a pneumatic tourniquet, the fracture was approached through a posteromedial lazy J incision, made with the longitudinal limb at the midline between the medial border of Achilles tendon and the posteromedial border of the distal tibia. The investing fascia was incised open, to expose the Achilles tendon and the avulsed bone fragment. The hematoma around the fracture was cleared, the calcaneal fracture was reduced and the bone fragment was fixed with a cancellous screw and a suture anchor, because the shape and thinness of the avulsed fragment did not allow the placement of a second screw. Satisfactory reduction of the fracture was achieved and the wound was closed in layers. Skin closure was performed with nylon sutures. Postoperative x-rays demonstrated adequate reduction of the fracture (Fig. **3**).

### 2.3 Primary Postoperative Course

Postoperatively, the patient was put in a plaster back slab with the foot in approximately 25 degrees of plantar flexion, so that no active ankle movement would be allowed and no stress applied on the Achilles tendon repair. Intravenous antibiotics were given for 48 hours and the patient was released from hospital 3 days post operatively, with instructions for daily subcutaneous low molecular weight heparin injections and a scheduled appointment for re-evaluation after a week.

### 2.4 Complication

**Fig. (4):**
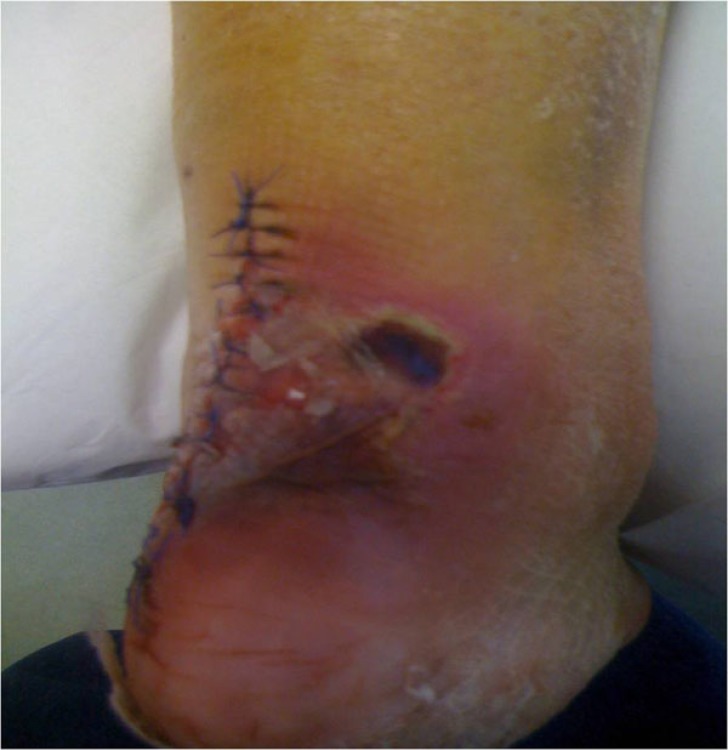
Skin necrosis and infection after re-fracture.

She failed to attend her scheduled appointment, and came back in the emergency department 2 weeks post-operatively, complaining of pain at the surgical site. She reported a new fall, when trying to walk around the house 5 days after the operation, but she didn’t return to the hospital immediately. The plaster was removed and a dark necrotic lesion 4x3cm was noticed, a few cm above and laterally to the surgical incision. A new lateral x-ray was taken and failure of the reduction was noticed, as the screw along with the bone fragment had been detached. Obviously the fragmented bone part had applied pressure to the overlying skin, causing local ischemia and necrosis. The skin around this necrotic area had the clinical appearance of infection, though we were unable to determine whether it was a local or a deep infection (Fig. **4**).

### 2.5 Revision Operation

**Fig. (7):**
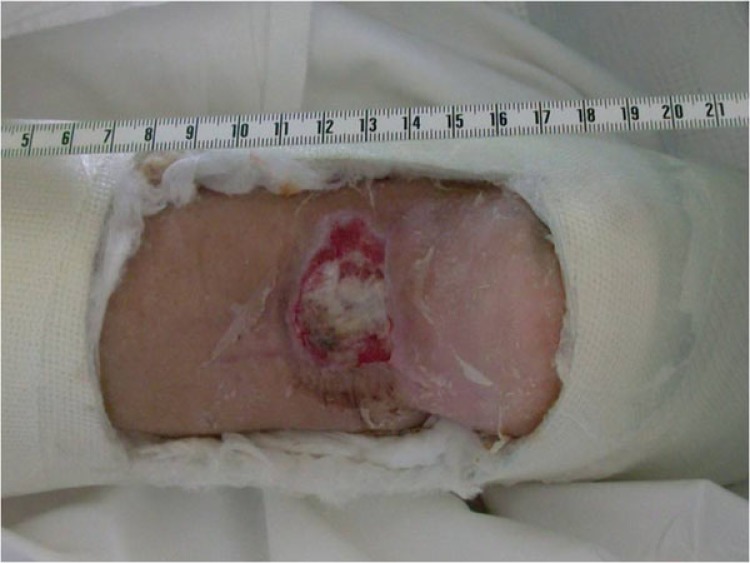
Plaster cast applied after debridement and tendon reattachment.

The patient was re-admitted and taken to the operating room for a second time. Since the soft tissue coverage of the area was inadequate and in the presence of infection, no further fixation was considered. Under local anesthesia and sedation, the necrotic skin was excised to healthy tissue. The bone fragment was removed together with the screw used for the initial osteosynthesis Fig. (**5**), but the attached part of the Achilles tendon was preserved and re-attached to the calcaneous tuberosity using two anchor sutures Fig. (**6**). The wound was inevitably left uncovered; a full plaster cast was applied, with the leg in plantar flexion and a posterior window to allow observation of the wound on a daily basis (Fig. **7**).

### 2.6 The Novel Technique

**Fig. (8):**
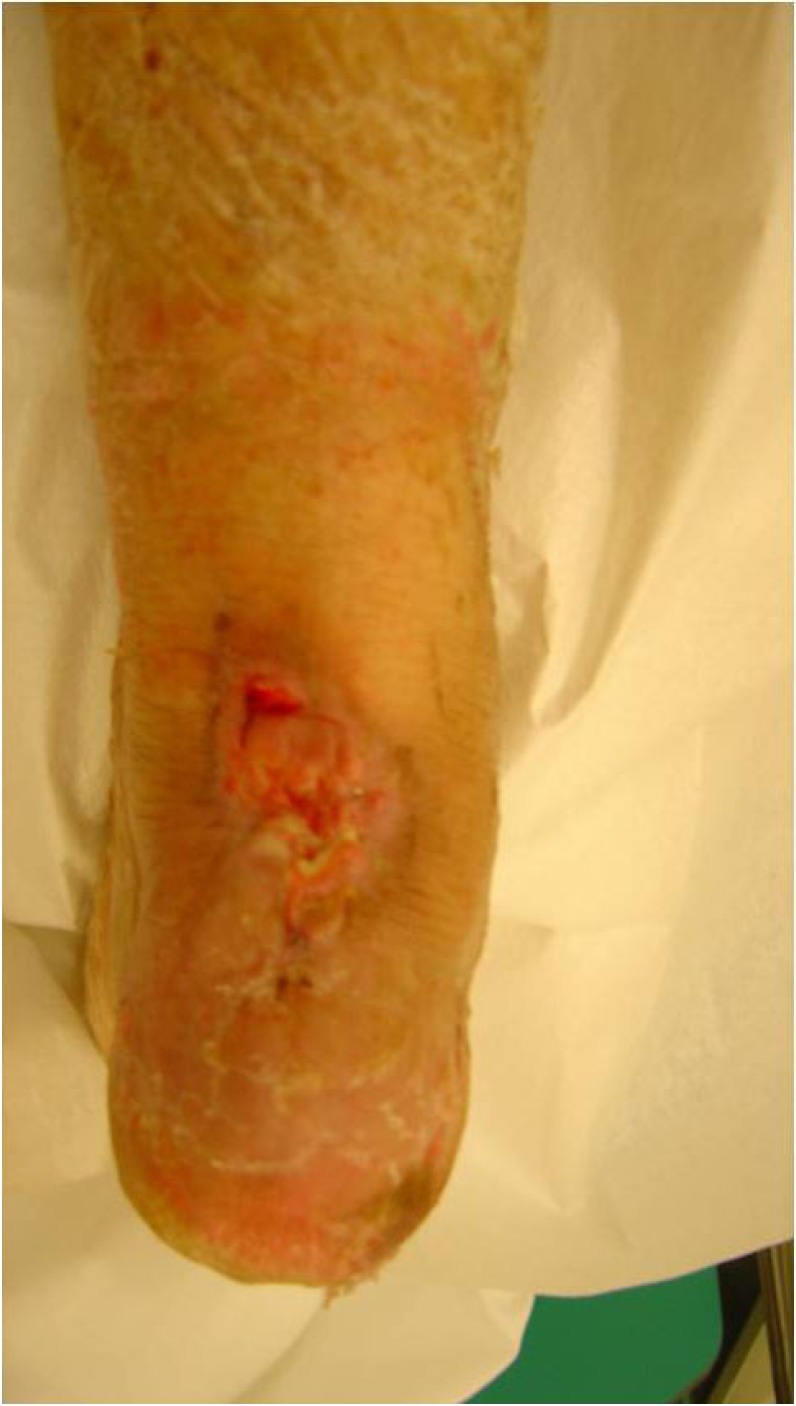
Wound before final coverage, eight weeks post op.

Platelet rich plasma (PRP) treatment was commenced to improve the soft tissue condition, reduce the size of the skin deficit and hopefully induce tendon-bone healing. Platelet rich plasma is an emerging biologic tool, that has been shown in dozens of published studies to improve healing of wounds, tendons and bone. Furthermore, V.A.C. therapy was locally applied. V.A.C therapy is the controlled application of sub-atmospheric pressure to a wound, using a therapy unit to intermittently or continuously convey negative pressure to a specialized wound dressing, to help promote wound healing and increase local angiogenesis. A total of 5 sessions of PRP treatment were applied along with the V.A.C. therapy, for a total period of eight weeks. The size of the skin lesion was reduced to 2x2 cm and the depth of the deficit was less than 1 cm (Fig. **8**).

### 2.7 Revision Postoperative Course

**Fig. (9):**
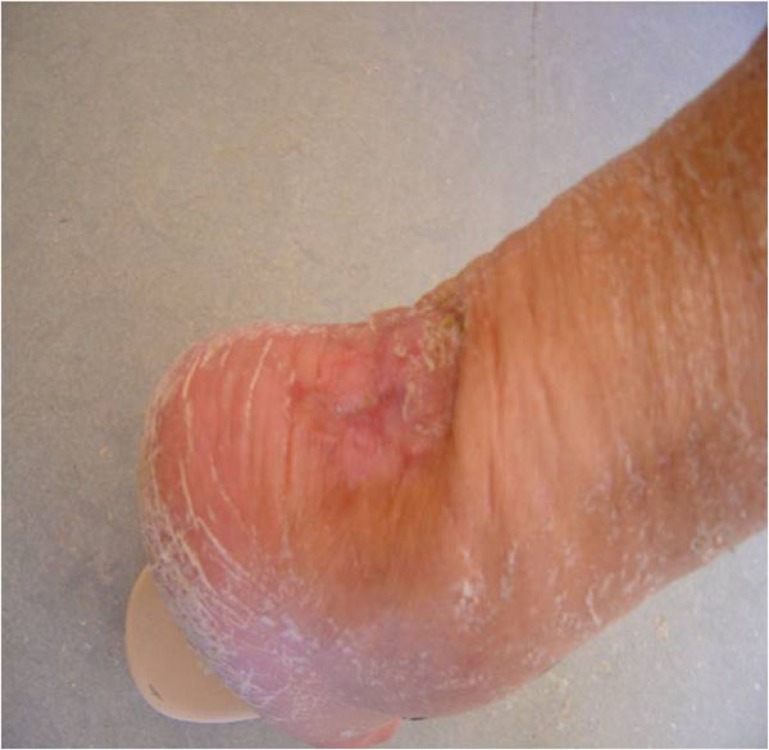
Complete, stable coverage, ten weeks post op.

**Fig. (10):**
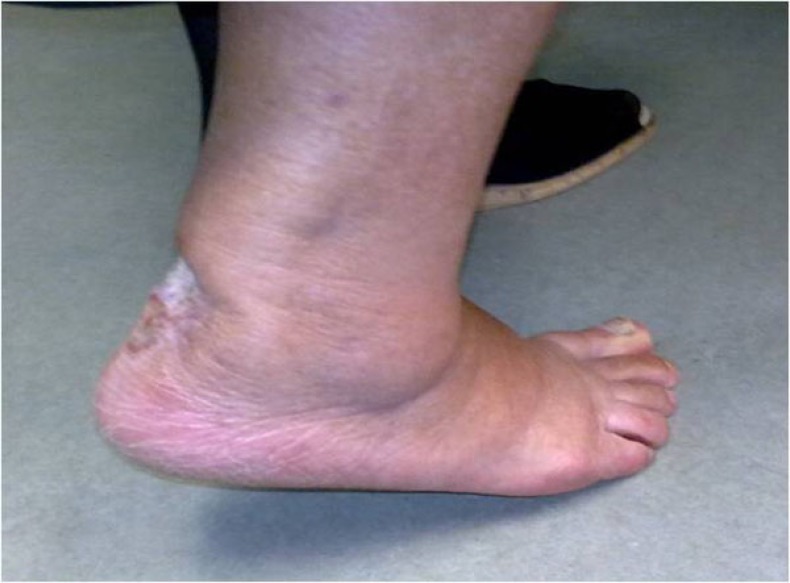
Final functional outcome, three months post-op.

At this point we decided to proceed with the coverage of the remaining lesion using a small rotational flap from the lower end of the wound, combined with full thickness skin grafts taken from the lateral left thigh. Two weeks later, the flap and 90% of the grafts had survived, the Achilles tendon was at that time completely covered (Fig. **9**) and the patient was put on a removable dynamic brace that allowed ankle mobilization and gradual weight bearing. Finally, 12 weeks after skin excision and tendon re-attachment the skin was completely healed, the patient had restored her right ankle motion (plantarflexion of 35 degrees and dorsiflexion of 15 measured with a goniometer) and was able to perform a single heel rise (Fig. **10**).

## 3 DISCUSSION

PRP, contains growth factors delivered from venous blood. The active growth factors are platelet derived growth factor, transforming growth factor beta, vascular endothelial growth factor, hepatocyte growth factor, fibroblast growth factor and epidermal growth factor. It is probable that a multitude of factors and cells play a role in inducing healing of hard or soft tissues that have been acutely or chronically injured or diseased. PRP can be used alone or in conjunction with surgical reconstruction to achieve better healing of tissues [5]. These growth factors aid healing by attracting un-differentiated cells in the newly formed matrix and triggering cell division [6]. PRP may suppress cytokine release and limit inflammation, interacting with macrophages to improve tissue healing and regeneration [7], promote new capillary growth [8, 9], and accelerate epithelialization [10] in chronic wounds.

Platelets in PRP also play a role in host defense mechanism at the wound site, by producing signaling proteins that attract macrophages [11]; PRP also may contain a small number of leukocytes [12, 6] that synthesize interleukins as part of a non-specific immune response. Previous studies of PRP have demonstrated antimicrobial activity against Escherichia coli, Staphylococcus aureus [13, 14], including methicillin-resistant Staphylococcus aureus [13], Candida albicans [14], and Cryptococcus neoformans [14]. A large number of studies have demonstrated the beneficial effects of PRP on the healing of tendons and ligaments at the molecular, cellular, animal, and human trail levels, but the efficacy of PRP treatment *in vivo* is still controversial in orthopaedics and sports medicine fields. Complex relationships among various molecules may be studied via the neutralization of individual or multiple growth factors. Although there are some studies in this direction [15-18], these relationships are still far from being clearly understood.

VAC, has been introduced in the management of complex wounds for its ability to remove third space fluids and improve oxygen delivery to the wound bed, while it promotes angiogenesis and granulation. VAC^®^ is a safe and effective method, facilitating delayed soft tissue reconstruction in complex lower limb traumas, in high risk patients. The development of healthy granulation tissue minimizes the need for major conventional reconstructive operations and therefore postoperative morbidity [19].

Skin necrosis after calcaneal avulsion type fractures [20], has mainly been attributed to delayed treatment [21] and despite being considered a dire complication there is no current consensus on optimal management mainly due to the lack of published data. [22, 23]. Supported treatment regimes include debridement with retention of hardware when possible, with antibiotic coverage up to flap coverage of the defect with Achilles re-advancement. However this data is mainly derived from case reports and subsequently lacks statistical power for specific recommendations to be made.The advantage of our approach rests in the functional restoration of the Achilles insertion and the ability to perform a wound debridement uncompromised by fixation and implant considerations, thus being able to work on healthy tissues. The combined application of PRP injection and VAC closure has only recently been described (well after the completion of our case treatment) in diabetic ulcers [24] and sternum osteomyelitis and sinus tract [25] with favorable results.

## CONCLUSION

Skin necrosis after calcaneal avulsion fractures, is a well known complication that can be even more difficult to treat if infected. Treatment by surgical excision of the necrotic and infected tissues including bone, followed by tendon stabilization with suture anchors and application of Platelet Rich Plasma and Vacuum Assisted Closure, in our case led to an excellent result.

## Figures and Tables

**Fig. (1):**
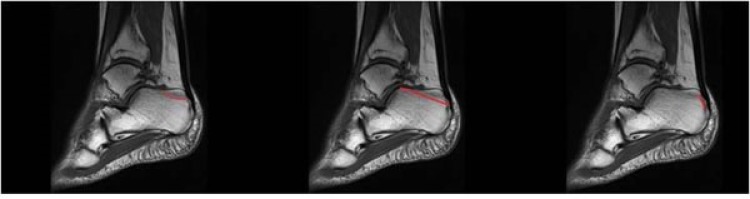
Rothberg classification of calcaneal avulsion fractures.

**Fig. (2):**
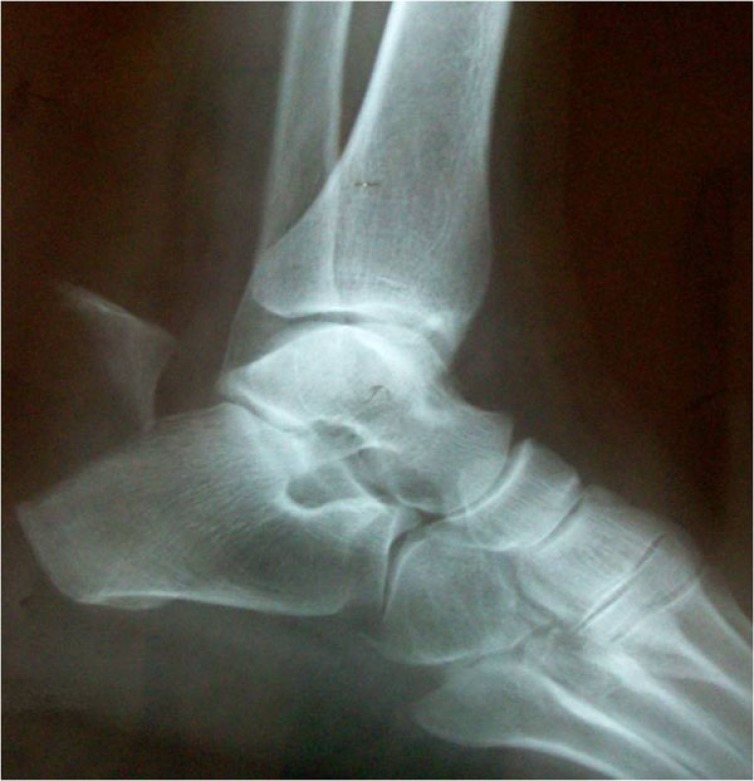
Lateral x-ray of the original fracture.

**Fig. (5):**
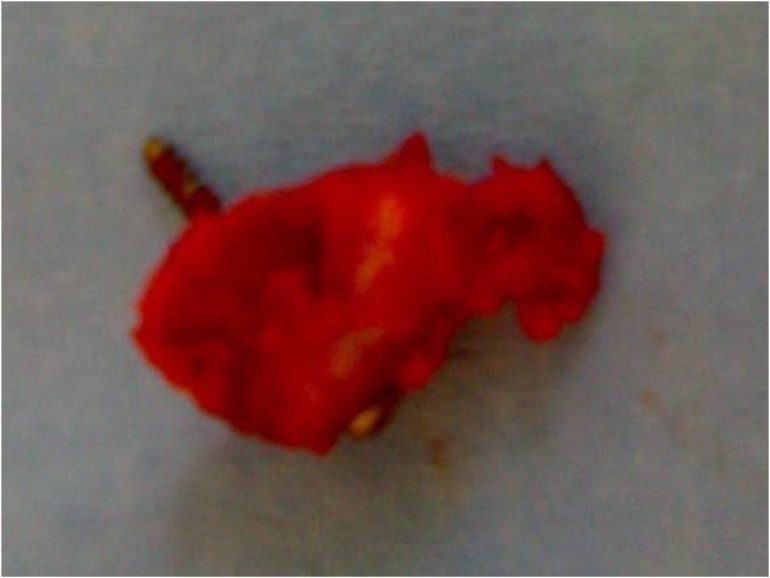
Removed bone fragment.

**Fig. (6):**
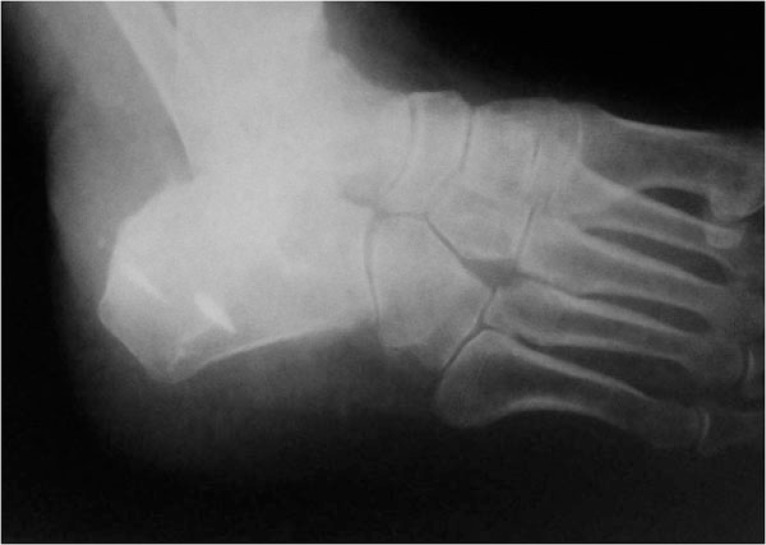
Lateral x-ray after reattachment of Achilles tendon with suture anchors.

## LIST OF ABBREVIATIONS

PRP: Platelet Rich Plasma
VAC: Vacuum Assisted Closure
